# In Vitro Determination of the Allergenic Potential of Egg White in Processed Meat

**DOI:** 10.1155/2010/238573

**Published:** 2010-03-17

**Authors:** Sabine Hildebrandt, Larsen Schütte, Stefan Stoyanov, Günther Hammer, Hans Steinhart, Angelika Paschke

**Affiliations:** ^1^Institute of Biochemistry and Food Chemistry, University of Hamburg, Grindelallee 117, 20146 Hamburg, Germany; ^2^Bundesforschungsanstalt für Ernährung und Lebensmittel, Institute of Meat Technology, E.-C. Baumann-Straße 20, 95326 Kulmbach, Germany

## Abstract

Hen's egg white has been reported as a causative agent of allergic reactions, with ovalbumin, conalbumin, ovomucoid, and lysozyme being the major allergens. However, little is known about the effects of processing with heat and high pressure on the allergenicity of egg white proteins as ingredients in meat. For this purpose, the allergenic characteristics of such treated preparations were studied. The IgE-binding capacity was analyzed by EAST inhibition in raw and processed meat preparations using sera from patients with hen's egg specific IgE. Increasing heat treatment as well as the application of high pressure decreased IgE binding, which is a measure of allergenic potential. The combined application of heat (70°C) and high pressure had synergistic effects in reducing the allergenic potential nearly twice as the sum of the single treatments conducted separately.

## 1. Introduction

Major allergens of egg white (ovalbumin, conalbumin, ovomucoid, and lysozyme), which rank among the most frequent initiators of food hypersensitivities in children and adults [1], are well characterized. The European Union project REDALL (Reduced Allergenicity of Processed Foods (Containing Animal Allergens), QLK1-CT-2002-02687), supports the development of technologies to reduce the allergenicity of products containing egg white. Many meat products contain additives and ingredients that may possess a risk for consumers in food allergies. This is especially true for egg which is used as a thickener in meat preparations. During food processing, the allergenicity of hen's egg may be altered by mincing and heating associated with industrial preparation of the final products. Moreover, chemical reactions during food processing between natural food ingredients and food additives can occur. Despite these potential sources of protein interaction, only a few allergens do not survive processing. Heat treatment has been recognized as a way of reducing allergenicity and boiled hen's egg has been reported as less allergenic [2–5]. However, severe food hypersensitivity reactions are also described for heat treated hen's egg [6]. Indeed, researchers have demonstrated the stability of ovomucoid against heat denaturation [7–11]. High pressure can also be used to denature proteins [12]. This study investigates the effects of heat and pressure on the binding of IgE to egg white proteins in processed food, particularly in meat preparations as an indication of allergenicity. 

A study accomplished by Schöberl [13] demonstrates that after high pressure treatment with >300 MPa raw meat samples showed an inactivation of enzymes, a consolidation of texture (caused by coagulation of dissolved sarcoplasmatic proteins), and a loss of the native red color (caused by autoxidation of myoglobine to brown metmyoglobine). However, the effect of high pressure on the allergenic potential of food is barely researched so far. Since studies by Jankiewicz et al. [14] and Scheibenzuber [15] showed alterations of the allergenic potential of different foods caused by >300 MPa and 600 MPa, respectively, the high pressure treatments progressed within the scope of these investigations were accomplished with 600 MPa.

## 2. Materials and Methods

### 2.1. Chemicals and Patient Sera

Phosphate buffered saline (PBS, 150 mM NaCl, 10 mM K_2_HPO_4_ at pH 7.4) was prepared as described by Bernhisel-Broadbent et al. [16]. If not otherwise mentioned, all chemicals were of analytical grade.

Patient sera were collected from 12 patients with egg allergy and a positive enzyme allergosorbent test (EAST, Spez. IgE ELISA RV 5, Allergopharma, Reinbek, Germany), class 2, 3, or 4 for egg white, and pooled. Patients were procured by the Technical University of Munich (Department of Dermatology and Allergology), the University Hospital of Zurich (Allergiestation, Dermatologische Klinik), the Macedonio Melloni Hospital of Milan (Department of Pediatrics), and the Medical University of Vienna (Department of Pediatrics and Juvenile Medicine). Two healthy patients without egg allergy were deployed as negative controls.

### 2.2. Samples

The processed meat matrix similar to a cooked sausage-batter with beef and fat from pork was established and provided by Bundesforschungsanstalt für Ernährung und Lebensmittel (Institute of Meat Technology, Kulmbach, Germany). Meat products were made from beef (shoulder, 56%), pork (back fat, 24%) and other technologically active substances. These are ice (18.3%), nitrite curing salt (1.65% NaNO_2_, in combination with 99.4 to 99.5% of table salt), dried egg powder (1%) and sodium ascorbate (0.05%). A sausage batter-system was used, which is near to industrial standards with the exception that besides fat no pork is used and that no spices are added.

Heat treatments were accomplished in the same way than it is usually done for the industrial production of fresh products (70°C) and different canned meat products (*F* = 1, *F* = 3, *F* = 12). Products like these are already on the market and well accepted by the consumer. The *F*-value is a measurement for the total quantity of heat that induces harmful effects for microorganisms. The *F*-value equals the time of heat treatment in minutes, which is required to reduce a specific bacteria count to an accepted end value [17].

**Figure 1:**
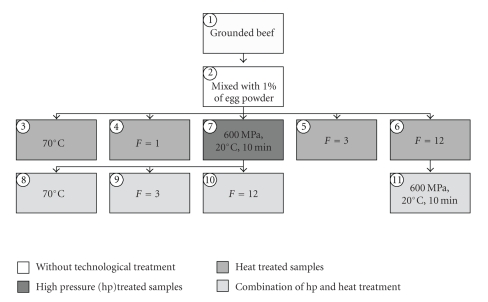
Steps of the technological process: (0): Standard: dried egg powder. (1): grounded beef and back fat of pigs. (2): chopped meat batches with 1% of dried egg powder. (3): heating of (2) to 70°C. (4): heating of (2) to an *F*-value of 1 (110°C, 2 atm). (5): heating of (2) to an *F*-value of 3 (110°C, 2 atm). (6): heating of (2) to an *F*-value of 12 (110°C, 2 atm). (7): high pressure treatment of (2) (600 MPa, 20°C, 10 minutes). (8): combination of (7) and (3) consecutively. (9): combination of (7) and (5) consecutively. (10): combination of (7) and (6) consecutively. (11): combination of (6) and (7) consecutively.

The following processing steps are demonstrated in Figure 1: (0): Standard: pasteurized dried egg powder (homogenized, pasteurized, spray-dried whole egg powder from chicken, provided by the company OVOBEST, Germany, EC-No: D/NI-EP-003/EWG). (1): grounded beef and back fat of pigs. (2): chopped meat batches with 1% of dried egg powder. (3): heating of (2) to 70°C (as in products labeled with “best before …” plus day and month, when still “best”). (4): heating of (2) to an *F*-value of 1 (110°C, 2 atm). (5): heating of (2) to an *F*-value of 3 (110°C, 2 atm, as in products labeled with “best before …” plus the year, when still “best”). (6): heating of (2) to an *F*-value of 12 (110°C, 2 atm). (7): high pressure treatment of (2) (600 MPa, 20°C, 10 minutes, in an autoclave). (8): combination of (7) and (3) consecutively. (9): combination of (7) and (5) consecutively. (10): combination of (7) and (6) consecutively. (11): combination of (6) and (7) consecutively. 

### 2.3. Protein Determination

Protein concentrations were determined relatively according to the method of Bradford [18], using bovine serum albumin as standard and Bradford reagent consisting of Coomassie Brilliant Blue G-250 and phosphoric acid.

### 2.4. Protein Extraction

Protein sample extracts have been carried out by mixing 1 g of the sample with 5 ml of PBS solution according to Leduc et al. [19] in a laboratory blender (Waring, New Hartford, USA) for 5 minutes. The homogenate was extracted for 1 hour on a laboratory shaker (Bühler, Tübingen, Germany) at 4°C and centrifuged for 30 minutes with 950 × g (Sigma, Osterode, Germany).

### 2.5. East Inhibition

EAST inhibition experiments are applied to compare the IgE binding potency of the different preparations. An additional homologous inhibition is carried out to check the allergenic activity of the native egg extract. The results are expressed as % inhibition. Extract potencies are quantitatively compared by estimating the protein concentration responsible for a 50% inhibition of the IgE binding to the solid phase (C_50_) from the inhibition graphs.

For EAST inhibition assay standard egg protein extract (PBS extracts see above) was linked to bromocyane activated paper discs (Schleicher & Schüll, Dassel, Germany) using a modified method from Ceska and Lundkvist [20]. 50 *μ*L of patients sera pool (diluted 1 : 2), previously incubated with different concentrations of the particular beef preparation or egg standard extract, were subsequently added to the discs and incubated for 3 hours at room temperature in cavities of a microtiter plate (Minisorb, 96 cavity, Nunc, Roskilde, Denmark). Allergopharma (Reinbek, Germany) test kit (Spez. IgE ELISA RV 5) was used for EAST inhibition according to the manufacturer's recommendations with modifications. Free binding sites were blocked with ethanolamine for 1 hour. Dilution series of the inhibitor extracts (extract (0) to (11)) were prepared in 7 steps (undiluted, 1 : 10, 1 : 100, 1 : 1000, 1 : 10000, 1 : 100000, 1 : 1000000). Potato protein was used to check nonspecific inhibition. A total of 50 *μ*L of diluted pool serum was added and incubated for 1 hour at 37°C in the dark. After 3 washes with 1% Tween 20 in PBS, 50 *μ*L of antihuman IgE alkaline phosphatase conjugate (Allergopharma, Reinbek, Germany, diluted 1 : 200 in incubation buffer) were added and incubated for 1.5 hours at 37°C in the dark. The plate was washed again and the bound enzyme activity was stained with 200 *μ*L of staining solution (containing p-nitrophenylphosphate (PNPP)) for 1 hour at 37°C in the dark. After the addition of stopping solution (100 *μ*L, 1 M NaOH) absorbance was measured at 405 nm. All EAST inhibition experiments were performed in duplicate and data were given in mean values.

## 3. Results

**Figure 2:**
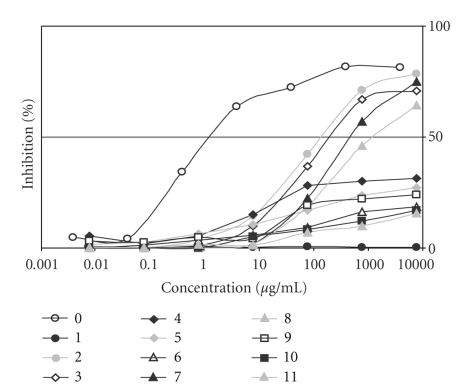
East inhibition with the different process step samples as inhibitors. (0): Standard: dried egg powder. (1): grounded beef and back fat of pigs. (2): chopped meat batches with 1% of dried egg powder. (3): heating of (2) to 70°C. (4): heating of (2) to an *F*-value of 1 (110°C, 2 atm). (5): heating of (2) to an *F*-value of 3 (110°C, 2 atm). (6): heating of (2) to an *F*-value of 12 (110°C, 2 atm). (7): high pressure treatment of (2) (600 MPa, 20°C, 10 minutes). (8): combination of (7) and (3) consecutively. (9): combination of (7) and (5) consecutively. (10): combination of (7) and (6) consecutively. (11): combination of (6) and (7) consecutively.

IgE binding of the protein extracts obtained after the different process steps was determined by the competitive EAST inhibition test using pool serum from hen's egg white allergic patients. The results are depicted in Figure 2 and the corresponding C_50_ values are shown in Table 1. The binding of IgE is an indication of potential allergenicity. In this assay, the degree of allergenicity was measured on the basis of inhibition of IgE by the total proteins in the extract from beef preparations. In this case, the higher the inhibition (means the lower the C_50_-value), the more allergenic the extract is. Results showed that—as expected—for the negative control (sample (1)) no C_50_-value could be measured. As sample (2) has a total egg white protein content of only 1%, it is expected that the C_50_-value of this sample should be 1% of that measured for the pure dried egg white powder (sample (0)). The results shown in Table 1 are in accordance with this expectation, as sample (0) has a C_50_-value of 1.3 *μ*g/ml and sample (1) a C_50_-value of 145 *μ*g/ml. An increasing intensity of heating of the samples resulted in lower inhibitions (and higher C_50_-values) which indicates a lower allergenic potential of the samples. Heating to 70°C (sample (3)) resulted in an increasing of the C_50_-value of 1.5fold of the unheated sample (2). After heating to an *F*-value of 1 the inhibition curve was such flat that a C_50_-value could not be measured with this EAST inhibition method. This is also considered for heated samples (5) and (6). 

High pressure treatment (sample (7)) resulted in an increasing of the C_50_-value of more than 3fold of the unheated sample (2). By additional heating to 70°C of the high pressure treated sample (sample (8)) the C_50_-value increased again of more than 2.5 fold in comparison to sample (7) that was high pressure treated only. The inhibition curves of the samples (9) to (11) (combinations of high pressure treatment and heating to an *F*-value of 3 or 12)) are flat and a C_50_-value could not be measured with this EAST inhibition method. Here, the inhibition curves of the samples with additional high pressure treatment are even more flat than that of the samples that are only heat treated. A comparison of samples (10) and (11) shows no obvious differences in the run of the curves whereas sample (11) that was at first heat treated with an *F*-value of 12 and than treated with high pressure tends to result in a slightly more flat curve than sample (10) where the treatings happened vice versa.

## 4. Discussion

For technological reasons—such as viscosity, cohesion, emulsification, and foam formation—whole egg powder is added to different foods during the manufacturing process. Leduc et al. [19] and Kato et al. [21] showed that the food matrix can influence the allergenic potential of the food ingredients during technological treatment. Therefore, within the scope of these investigations the allergenic potential of a model food was examined. For that purpose a meat preparation with an additive of powdered whole egg (see experimental section) for the production of cooked sausages was used as a model food. For technological treatments different high pressure and/or heat treatments were used (see experimental section). 

The results of the investigations of the powdered whole egg used as an additive in meat preparations for the production of boiled sausages showed that an increasing heat treatment as well as a high pressure treatment results in a clear alteration of the egg proteins and their allergenic properties. Studies showed that the longer the heating time, the stronger the reduction of IgE-binding properties, which is in accordance with the findings of this study [22–24]. After heat treatment of the sample with 70°C a reduction of the allergenic potential of 1.5fold in comparison to the unheated sample could be measured by EAST inhibition method. All other heat treatments (F = 1, F = 3, F = 12) resulted in a very strong reduction of the allergenic potential in such a way that it could no longer be measured by EAST inhibition method. Nevertheless, a tendency of the decreasing of the allergenic potential from F = 1 through F = 3 to F = 12 can be derived from the curve progressions of the EAST inhibition method (Figure 2). EAST inhibition results also showed that high pressure treatment of the sample caused a 3.3fold reduction of the egg allergenic potential in comparison to the untreated sample. Thus high pressure treatment resulted in a 2.5fold stronger reduction of the allergenic potential of the egg sample than heat treatment to 70°C, but clearly weaker as all other heat treatments (F = 1, F = 3, F = 12). However, a high pressure induced increasing of antigenicity of beta-lactoglobulin accomplished by ELISA methods with specific monoclonal hen's antibodies could be shown by Illgner [25]. This can be referred to high pressure induced increase of the protein surface but it is not known if these results have clinical relevance. Nevertheless, these different results demonstrate that the effects of high pressure treatment through the allergenic potential of food can be different for diverse allergens in diverse matrices. By the combination of heat and high pressure treatment a higher reduction of the allergenic potential of the sample could be accomplished than caused by the single treatments. The high pressure treatment of the sample that was preheated to 70°C resulted in a 8.9fold stronger reduction in comparison of the untreated sample. Consequently it became clear that the combination of both treatments caused a nearly 2fold stronger reduction of the allergenic potential as the sum of the single treatments conduced separately (1.3fold + 3.3fold = 4.6fold). For all other combinations of heat and high pressure treatments these synergistic effects could not be measured in detail as the C_50_-values of the EAST inhibition method could not be quantified because of the very low allergenic potential of the samples. To investigate if the order of the technological treatments has an influence of the allergenic potential EAST inhibition curves of samples (10) and (11) (see Figure 2) were compared. Here, no significant differences in the allergenic potential could be shown. Nevertheless, the reduction of the allergenic potential tends to be a little stronger for sample (11) that was at first heat treated and than treated with high pressure. 

The sensitivity of the EAST inhibition assay is highly dependent on the used patient's sera. In this case a reduction of the allergenic potential of about 1000fold compared to the native egg extract could still be measured as elucidated in Table 1.

## 5. Conclusions

The aim of this study was to determine whether heat, high pressure, or a combination of both treatments influences the allergenicity of hen's egg white allergens in meat preparations. Heat treatment with an *F*-value of 3 or 12 brought about a significant decrease in allergenicity measured by EAST inhibition. Heat treatment with 70°C caused a decrease of about 1.5fold in allergenic potential measured with EAST inhibition. High pressure treatment resulted in reductions in IgE-binding properties of more than 3fold. The combined application of heat (70°C) and high pressure produced synergistic effects reducing allergenic potential by nearly twice as much as the sum of the single treatments conducted separately.

## Figures and Tables

**Table 1:** C_50_-values of the EAST inhibition experiments.

Sample no.	C_50_-value (*μ*g mL^−1^)
0	1.3
1	–^a^
2	145
3	214
4	–
5	–
6	–
7	485
8	1286
9	–
10	–
11	–

^a^No C_50_-value could be measured.
